# A Remarkable Case

**Published:** 1891-02

**Authors:** 


### A REMARKABLE CASE.

One of the strangest cases to the medical profession, is that of Miss Grace Gridley, a young lady of Amboy, Ill. Her singular case has engaged the attention of the medical profession far and wide, and they have thus far been unable to solve the mystery. Her mother says : Grace had a severe case of la grippe along in February, from which we supposed she had fully recovered, but in March she had a relapse, which left her languid. She was always a bright, active child of a nervous temperament. She is now over twenty-two years of age. We first noticed that, contrary to her usual custom, she wanted to lie in bed in the morning, which gradually grew upon her until on the 1st of April we were unable to arouse her.

Medical assistance was called and not until a strong current of electricity had been applied did she awake. She remained awake for two days, and on the third we again had to resort to electricity. She awoke with a scream and said: "Don't do that; it hurts me so. Mamma, I don't want to wake; I am so comfortable. I understand and know every thing that is going on around me," and she sank into that dreadful lethargy you find her in to-day. Yes, she got out of bed once. It was several days after the second application of the battery, and we found her in the front parlor—her room is upstairs—by the window with an open Bible on her lap. She turned the leaves restlessly, but did not seem to read. I am sure she did not know what she was doing, and she did not seem to recognize me when I led her back to her room. She seemed conscious at times, and to this day seems to notice the presence of a stranger in the room, as the presence of any but the family seems to distress her.

When asked if it had not been reported that the strange illness had been brought on by religious excitement at a revival, Mrs Gridley said: There has been such a report, but there is no truth in it, though Grace is an ardent Christian and a member of the church. She has not attended any revivals for a year, and was never unduly excited over religion.

Miss Gridley is slowly wasting away, though she is given liquid food about every three hours, and swallows naturally. At times she seems to be awake, opening her eyes about a quarter of the way. She turns herself in the bed, and seems to have control of her limbs. "When she has opened her eyes," said Mrs. Gridley, "we have earnestly asked, even implored, Grace to make some sign, a movement of hand or eye, or to do something to show that she recognizes us, but she makes no sign. We can't understand it."—*St. Louis Republic.*

